# Three-Rooted Permanent Mandibular First Molars: A Meta-Analysis of Prevalence

**DOI:** 10.1155/2022/9411076

**Published:** 2022-03-28

**Authors:** Nyan M. Aung, Kyaw K. Myint

**Affiliations:** ^1^Department of Medical Services, Ministry of Health, Mandalay, Myanmar; ^2^Department of Oral Biological Science, University of Dental Medicine (Mandalay), Mandalay 05041, Myanmar

## Abstract

**Introduction:**

Although numerous amounts of high-level evidence were present, they solely emphasized the tooth-level prevalence of three-rooted permanent mandibular first molar. Global patient-level prevalence and bilateral symmetrical distribution of this type of teeth were needed to be tackled across the world. The research question was “What is the global prevalence of three-rooted permanent mandibular first molars?”

**Materials and Methods:**

In vivo epidemiological studies undergone with Cone Beam Computed Tomography (CBCT) were eligible. The proportions of the prevalence of three-rooted permanent mandibular first molars were presented in the forest plots by random effect model. The calculation was performed with MetaXL version 5.3. Subgroup analysis, sensitivity analysis, and publication bias method were also calculated.

**Results:**

Seventy-two studies from 31 countries were selected for both qualitative and quantitative analyses. 26302 patients and 37994 permanent mandibular first molars were included in the analysis. 9% of permanent mandibular first molars all over the world demonstrated 3 roots. These three-rooted teeth were found in 10% of the world population, more than 45% of which revealed bilateral symmetry of that anatomy. Right-side dominance and no sexual dimorphism were seen in the distribution of three-rooted permanent mandibular first molars. Global tooth-level prevalence of Radix Entomolaris and Radix Paramolaris was 12% and 0.1%, respectively.

**Conclusion:**

The prevalence of three-rooted permanent mandibular first molars (PMFMs) was influenced by different geographical locations across the world and also by widespread habitation of the Mongoloid descent. The authors postulate that globalization, together with blending among ethnicities, may have a great impact on the reduction or accentuation of the anatomical significance in some populations.

## 1. Introduction

**Figure 1:**
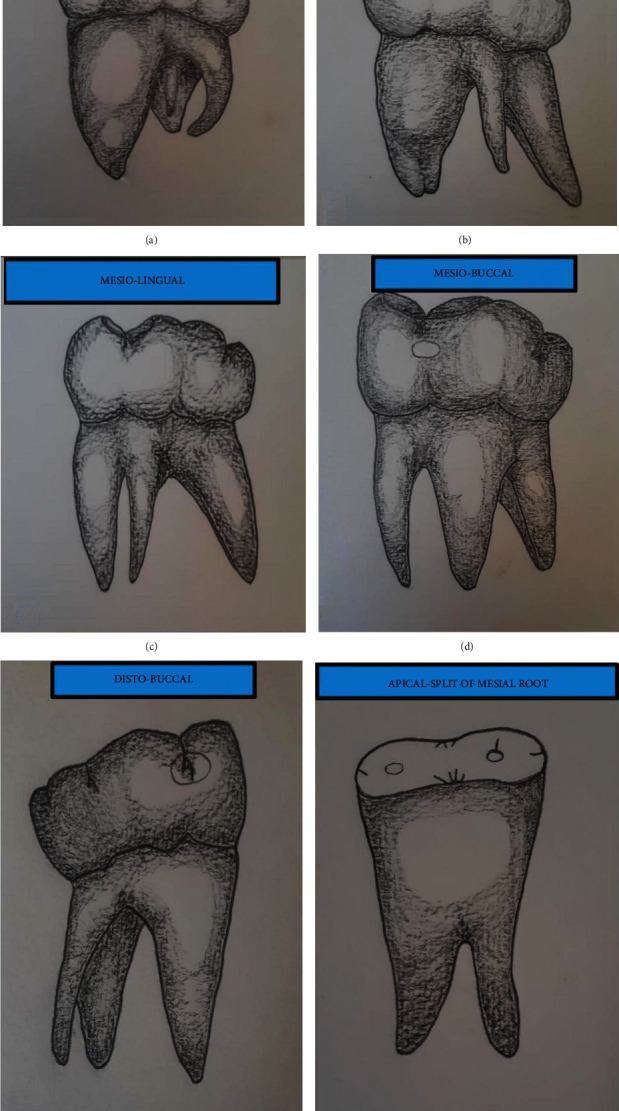
Variations in the position of the third root of right PMFM: (a) distolingual position at which the third root stands from the lingual aspect of the distal root, (b) centrolingual position at which the third root is midway between mesial and distal roots at the lingual surface, (c) mesial-lingual position at which the third root branches from and lingual to mesial root, (d) mesiobuccal position at which the third root stems from buccal aspect of mesial root, (e) distobuccal position at which the third root rises from buccal to distal root, and (f) the third root that splits from the apical third of the mesial root.

The term “three-rooted permanent mandibular first molar (PMFM)” is more generalized and less specific than others, based on the counting number of roots. So, most of the researchers used the more famous one, “Radix Entomolaris (RE),” instead of the former. “Radix” means “root” [1], and “Ento” denotes “Inside” [1], both of which in turn can be understood as “the root merging from the lingual side.” Comparatively, Radix Entomolaris can be stated as the accessory root originating from the lingual root trunk of the molar. Another word “Para” means “Beside” [1], which can be recognized as “buccal side of the mouth.” As a result, Radix Paramolaris (RP) can be defined as “a root originating from the buccal side of the molar.” Both of the two terms are constituted as three-rooted PMFMs. Recently, some evidence found that there could be many positions of an accessory root along the root trunk of PMFM, frequently distolingual or centrolingual and rarely distobuccal third roots [2–4] (Figure 1). However, the American Association of Endodontists (AAE) [5] recorded RE as a distolingual root and RP as a mesiobuccal one. Although definitions of the conditions should further be modified and more meaningful, we used the more generalized one, “three-rooted PMFM,” in our present meta-analysis.

From a clinical perspective, radix mandibular first molars display some significant features. Up to 32% of these teeth showed an additional tubercle or sixth cusp in its occlusal anatomy [6] in contrast to normal five-cusped crown morphology. They had averaged 0.3 mm wider in buccolingual measurement at the distal surface of the crown [7] than that of its two-rooted counterparts. Additionally, intercuspal distances between the distolingual cusp and the adjacent distobuccal and mesiobuccal cusps were slightly wider in three-rooted PMFM than in two-rooted one [7].

Buccolingual curvature of the third root of three-rooted PMFM comprised more than 30 degrees measured by Schneider method [8]. Nearly 60% of radix roots displayed this severe curve [8]. Mesiodistal curvature of these roots was less prominent than the buccolingual curve [8]. Some investigators found that an excessive degree of curvature was the origin of the separation of the rotary endodontic Ni–Ti files [9]. One systematic review figured out that the thermoplasticized method was superior in adaptation between root canal wall and gutta-percha than the lateral condensation method [10]. Most of the investigators of primary studies in this review postulated that the method may be suitable for complex anatomy such as high curvature.

**Figure 2:**
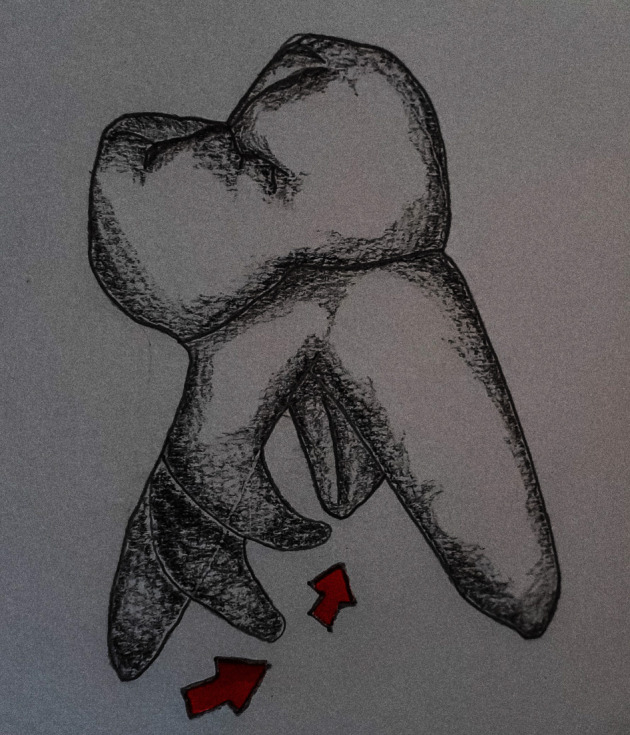
Distal proximal view of right three-rooted PMFM showing various curvatures of the distolingual root.

Straight extra root could be more readily overlapped by distobuccal root than the curve one in periapical X-ray (PA) [11]. Consequently, the curvature of the distolingual root canal was more prominent in the proximal view than in the clinical view (PA view) [11]. In proximal view, buccolingual curvature was classified into straight, coronal curve, and apical curve in the buccolingual plane [12] (Figure 2).

**Figure 3:**
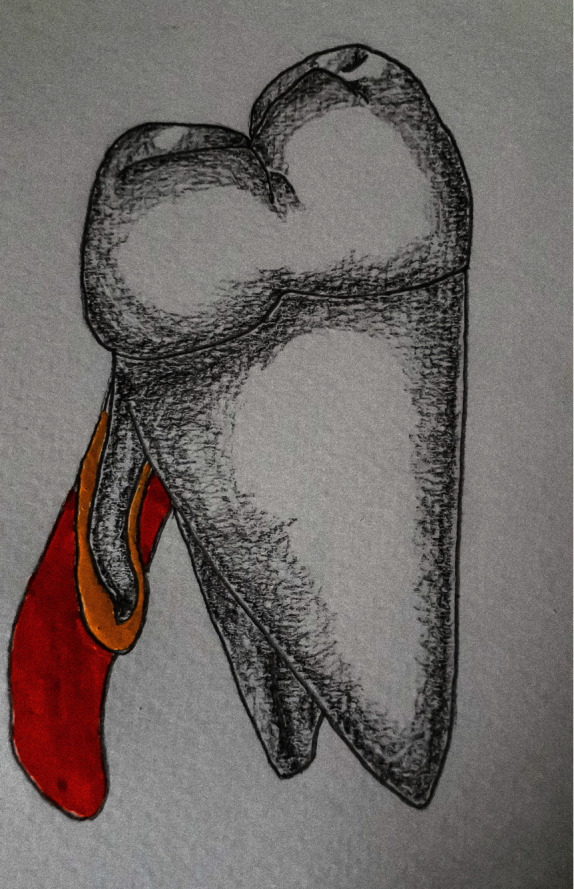
Distal proximal view of right three-rooted PMFM showing variations in the size of the distolingual root.

The size of the distolingual roots may vary from short conical structure to normal root length up to 8 mm from cervix to apex [12] (Figure 3). Sometimes due to its tiny dimension, there was more apical structure from distobuccal and mesiobuccal roots that needed to be resected to access distolingual root during endodontic microsurgery [13].

Trifurcation, resulting from the presence of the extra root, was located approximately 1 mm apical to mesiodistal furcation [14]. This may worsen severe periodontitis in comparison with its two-rooted counterpart [15]. Surprisingly, the point of separation of Radix Paramolaris (RP) was commonly detected at the apical third of the root in an Israeli population [2] (Figure 1(f)).

Anatomy of the pulp chamber floor was switched from a normal triangular pattern in two-rooted PMFMs to trapezoidal in three-rooted PMFMs [4]. The orifice of the extra root was located approximately or more than 3 mm lingual to distobuccal orifice [8]. However, excessive deposition of secondary dentine could unite the roof and floor of the pulp chamber, thereby interfering with the orifice of the distolingual root. Intricate root canal anatomy was seen up to 26% of radix root [4]. RE usually has one canal with a nearly circular cross section [4].

Bilateral occurrence of PMFMs with distolingual roots was positively associated with the increase in the prevalence of the complicated root canal in mandibular lateral incisors [16] and the presence of an additional root and C-shaped root canal anatomy in mandibular first premolars in the Taiwanese population [17]. These studies were done by the CBCT in vivo method.

The empirical in vivo study applied the extraction method in the assessment of RE [18]. Nowadays, the method is not suitable for current clinical practice. And patient-level prevalence and bilateral symmetry of three-rooted PMFM cannot be identified by the extraction method. Then, this type of research was replaced with an in vivo X-ray methodology. Some investigators [19] suggested that two radiolucent lines, corresponding to root canal and periodontal ligament of an additional root, were diagnostically detected in PA view. This may be seen crossing the distobuccal root in the view. However, it needs 25–30-degree mesial horizontal angulation of the X-ray beam to capture RE [11]. Although some investigators used the X-ray method correctly, others confirmed RE only in one sagittal plane X-ray. One study argued that almost 60% of RE was sensitively detected in PA view [11]. Nowadays, micro-CT is a gold standard, which needs a priori extraction. But, the surgery cannot guarantee the preservation of intact root structure. So, this can lead to the liberation of underestimated and nongeneralizable findings. Finally, in vivo CBCT research methodology is a convenient solution to tackle the incidence of three-rooted PMFM in both research and clinical diagnosis. The axial slice of CBCT can ease the reliability to count the number of roots in PMFM. The data from such methodology could be readily recorded, available for research, and retrospectively extracted from both databases of public and private sectors.

The growing evidence demonstrated the incidence of the additional root in different types of teeth. The accessory root revealed approximately 0.8% at the palatal root of permanent maxillary first molars in the Greece-Turkish population [20], around 8% in permanent mandibular second premolars of the Indian population [21], up to 12% in permanent mandibular canines of the Iranian population [22], and almost 16% in permanent mandibular first premolars of the African-American population, respectively [23]. For PMFM, an earliest systematic review of the scope investigated that the pooled prevalence of three-rooted PMFM revealed 13% with the highest value of 22% in the Chinese population [24].

The recent systematic reviews of the specific populations pointed out that the incidence of three-rooted PMFM demonstrated up to 3% in Brazil [25] and almost 6% in Saudi Arabia [26]. One systematic review [27] investigated that the pooled prevalence of 3-rooted PMFMs was 8.9% resulting from the meta-analysis of 35 studies across the world. Although a huge amount of high-level evidence is currently being published in the scope of endodontic anatomy, they solely emphasized the tooth-level prevalence of three-rooted PMFMs. And some researchers used the studies of different research methodologies, pooling both in vivo and in vitro results. This may harm the consistency of the pooled estimate.

As a consequence, patient-level prevalence and bilateral symmetrical distribution of three-rooted PMFMs were needed to investigate across the world. Additionally, different frequency of the prevalence across Asia's regions was still on demand, although a single systematic review for Saudi Arabia intervened recently. In addition, RE is more clinically significant than RP. Curiously, researchers and clinicians usually mark the prevalence of RE. For that reason, there was a lack of evidence pooling the prevalence of RP, which also has anatomical significance. Both RE and RP can be postulated as the term “three-rooted PMFM.”

The research question of the present meta-analysis was “What is the global prevalence of three-rooted PMFMs?”

The objectives were as follows:

- To investigate the global tooth-level prevalence of three-rooted PMFMs.
- To estimate the global patient-level prevalence of three-rooted PMFMs.
- To detect global patient-level bilateral symmetry of three-rooted PMFMs.

## 2. Materials and Methods

### 2.1. Selection Criteria

#### 2.1.1. Type of Included Studies

The studies eligible for inclusion were as follows:

1. In vivo.
2. Undergone by means of Cone Beam Computed Tomography (CBCT) or Spiral Computed Tomography (SCT) methodology.
3. Cross-sectional.
4. Prospective or retrospective.
5. Analytical or descriptive.
6. Primary or secondary objectives, including “number of roots” or the term inferred from the variations in the number of roots.

#### 2.1.2. Characteristics of Excluded Studies

The features of excluded studies were as follows:

1. Studies that did not report the age of the patients or developmental condition of the root apex
2. In vitro
3. Methods other than CBCT or Spiral CT
4. Primary or secondary objectives not including “three roots” or “number of roots”
5. Studies that did not present “the number of roots”
6. Studies used more than one research methodology (e.g., CBCT + Periapical X-ray)
7. Secondary data analysis, book chapter, case reports, narrative reviews, editorials, opinions, letters to the editor, animal studies, and personnel communication

#### 2.1.3. Target Conditions (Numerator Variables)

1. Three-rooted PMFM (objective Ι, subgroup analyses ΙV and V, and sensitivity analysis VΙ)
2. Patient with three-rooted PMFM (objective ΙΙ)
3. Patient with the bilateral presence of three-rooted PMFMs (objective ΙΙΙ)

#### 2.1.4. Population

Patient with permanent mandibular first molars (PMFMs) that had no external (or) internal resorption, no other root anomalies, fully formed root apex and the age, which was compactible to completed root development, was included.

The denominator variables were as follows:

1. Total number of teeth (PMFM) for objective Ι, subgroup analyses ΙV and V, and sensitivity analysis VΙ (tooth-level prevalence)
2. Total number of patients for objective ΙΙ and the total number of patients with three-rooted PMFMs for objective ΙΙΙ (patient-level prevalence)

### 2.2. Search Strategies

The literature was searched in the frame of condition, context, and population. The search words were as follows:

1. *Condition*. Three roots, three-rooted, 3-rooted, 3 roots, third root, three separate roots, distolingual root, two distal roots, extra distal root, extra distolingual root, DL root, DLR, DL, extra DL, extra root, additional root, supernumerary root, 2R2C, Radix Entomolaris, Radix Molaris, Radix Paramolaris, radix first molars, 3RM1, and radix molars
2. *Context*. CBCT, Cone Beam Computed Tomography, Cone Beam CT, Spiral Computed Tomography, and Computed Tomography
3. *Population*. Permanent mandibular first molars, permanent lower first molars, mandibular first molar, lower first molars, lower molar, mandibular molars, lower posterior teeth, mandibular posterior teeth, mandibular 1st molar, lower 6, and PMFM

The search strategies were decorated through 1 AND 2 AND 3 through the selected databases. PubMed, Goggle Scholar, Research Gate, ProQuest, and LILACS were included. There was no language and time restriction. Searching was undertaken until October 30, 2021. Magnifying from reference lists of the articles and Goggle Search was also done.

### 2.3. Data Collection

We accessed the formerly stated data for each study: sample characteristics (total number of PMFMs in the study sample, sample size (patients), number of three-rooted PMFMs, patients with three-rooted PMFM, bilateral symmetrical distribution of three-rooted PMFM in patients with PMFMs on both sides of the mandible, total number of teeth in male and females patients, total number of teeth on left and right sides of the mandible, total number of Radix Entomolaris and Radix Paramolaris, gender, age, geographical locations, country, and ethnicity), study characteristics (sampling frame, randomization, sample size calculation, and statistical analysis), and setting (CBCT scan, voxel size, field of view (FOV), mA, slice thickness, kVp, type of viewer software, type of examiners, and reliability test).

Manuscripts of some studies were translated from native languages to English by using the Mobile Application of Goggle Translate.

### 2.4. Assessment of Methodological Quality

We assessed the methodology of the selected studies by the Joanna Briggs Institute (JBI) Critical Appraisal tool for systematic reviews of prevalence studies. The appraisal tool contains nine questionnaires. Of these, the 9th question, which is routinely used to assess the response of the participants, was irrelevant for this meta-analysis and so was excluded. As a result, we attempted 8 JBI questionnaires. The selected articles were assessed and subgrouped as high risk of bias (JBI score ≤49%), moderate risk of bias (JBI score ranging from 50% to 69%), and low risk of bias (JBI score >70%) [28].

Together with the methodology quality assessment, the following domains of research methodology of included studies were subjectively analyzed, then listed, and graphed: sampling frame, randomization, sample size calculation, complete reporting of context and settings, reasonable population coverage, validity of measurement, reliability of measurement, complete outcome reporting, and appropriate statistical analysis. This was done by transforming the original data to proportion and then graphing.

### 2.5. Statistical Analysis

For the descriptive and qualitative purpose, the JBI scores were added together by the influence of the 8 questions mentioned above and transformed into proportions. The three formulas were as follows: (No. of Three − rooted PMFMs/Total no. of PMFMs) × 100 for objective Ι. (No. of Patients with three − rooted PMFMs/Total no. of Patients) × 100 for objective ΙΙ.(No. of Patients with bilateral three − rooted PMFMs /Total no. of Patients with three − rooted PMFMs) × 100 for objective ΙΙΙ.

Occasionally, patient-level prevalence data, which was not reported in primary studies, was back-calculated from unilateral and bilateral distributions of three-rooted PMFMs.

The proportions of the prevalence of the individual study were calculated and presented in the forest plots by random effect model. The estimation was calculated in an Excel spreadsheet and MetaXL version 5.3. Data extraction and back-calculation were undertaken whenever direct usage of the data was not possible from primary studies. *P*-value was agreed upon as 5%.

Tests for heterogeneity were *Q* and *I*2 statistics. *I*2 is the calculation of between-studies heterogeneity. The final results were pooled as global tooth-level prevalence of three-rooted PMFM, global patient-level prevalence of three-rooted PMFM, and global patient-level bilateral symmetry of three-rooted PMFM (objectives Ι, ΙΙ, and ΙΙΙ).

To explore heterogeneity, subgroup analyses were done through the prevalence of three-rooted PMFM according to different geographical locations across the world in addition to the pooled estimates of objectives Ι, ΙΙ, and ΙΙΙ. Additionally, subgroup analyses were undergone with regard to left and right and gender distributions (subgroup analyses ΙV and V).

To assess the pooled estimates of the tooth-level prevalence of RE and RP, sensitivity analysis (VΙ) was undergone by the exclusion from the selected studies which did not report “Radix Entomolaris,” “Radix Paramolaris,” and the terms matched with “RE” and “RP.”

### 2.6. Publication Bias Methods

Visual inspection of funnel plot asymmetry was the test for publication bias in the review. The *x*-axis of the funnel plot was set as double arcsine prevalence. Arcsine transformation was needed with the data of extreme values such as 0 or 1. Otherwise, values, out of the range of 0 and 1, which mean 0% and 100%, could be included in the confidence interval of the proportion [29]. Precision resulting from the inverse of Standard Error (SE) was set at the *y*-axis of the plot.

In MetaXL, funnel plot asymmetry was confirmed by Doi plot and LFK index for publication bias. Doi plot indicates “no asymmetry” (no publication bias), “minor asymmetry” (minor publication bias), and “major asymmetry” (major publication bias). Beyond ±1 of the LFK index describes the presence of publication bias [29].

## 3. Results

The proposal of the present meta-analysis was registered in PROSPERO and available at https://www.crd.york.ac.uk/prospero/display_record.php?ID=CRD42022302195. The registration number is CRD42022302195.

**Figure 4:**
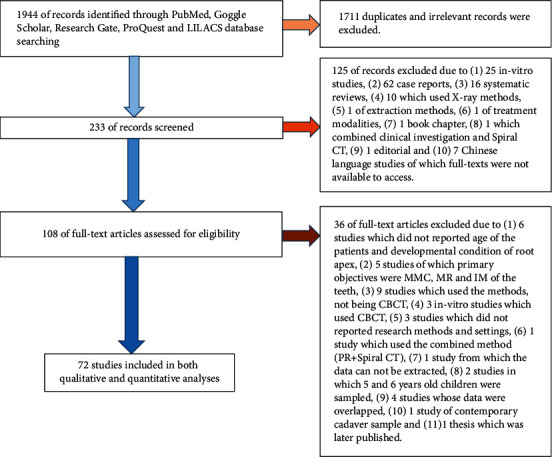
Flow diagram of identifying, screening, and processing the studies.

Selection and exclusion of the records were demonstrated in PRISMA (Preferred Reporting Items for Systematic Reviews and Meta-Analyses) flow diagram (Figure 4). A total of 72 studies were selected for both qualitative and quantitative analyses.

Qualitatively, 10 studies presented a JBI score of 5/8, 27 studies a score of 4/8, 24 studies 3/8, 7 studies 2/8, and 4 studies 1/8. As a result, we divided these into two categories: JBI scores 4 + 5 and ≤3. Thirty-seven studies obtained an average JBI score of 53.38%, indicating a moderate risk of bias, and 35 studies obtained the average score of 32.14% comprising high risk of bias.

**Figure 5:**
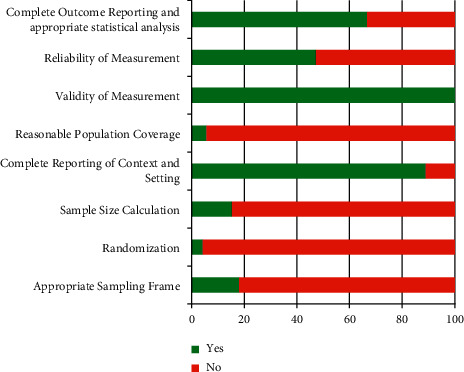
Summary of research methodology of the included studies.

The reported research methods of all included studies were categorized in Figure 5.

Table 1 presents the global prevalence of three-rooted permanent mandibular first molars with population, country, number of patients, number of teeth, geographic location, the condition termed in primary studies, settings, and study design.

26302 patients were included in the systematic review, of which 10003 were males and 11242 were females. Seventeen studies enclosed the gender status of study participants.

37994 permanent mandibular first molars were involved in this review. Four studies did not present the number of teeth. The number of patients with three-rooted PMFMs was included in the objectives of these studies.

A cross-sectional descriptive retrospective design was applied in 58 studies, 13 cross-sectional analytical retrospective studies, and only 1 cross-sectional analytical prospective study.

Nineteen studies presented that their outcome of interest was similar to the primary objectives (three-rooted PMFM) of the current meta-analysis. The remaining 53 studies showed “the number of roots” as a secondary objective.

Nine studies were conducted in South Korea, 8 in China, 6 in Turkey and Saudi Arabia, respectively, 5 in Taiwan and Iran individually, 4 in Brazil, 3 in Malaysia, and 2 in India, Portugal, and Chile particularly. Only one study was individually selected from Hong Kong, Japan, Egypt, South Africa, United States, Italy, Belgium, Serbia, Spain, France, Russia, Greece, Pakistan, Nepal, Thailand, Vietnam, Iraq, UAE, Yemen, and Israel.

Sixty-eight studies were published in English. Out of these, 4 studies were translated from native languages to English. Of these, 1 study from China was translated from Chinese to English, 1 Iranian study from Kurdish to English, 1 Saudi Arabia study from Arabic to English, and 1 from Japanese to English.

One study compared Portugal and Chinese populations. One study was conducted with both Belgium and Chilean populations. One study used both Saudi Arabian and Indian samples for comparative purposes.

Three-rooted PMFMs were not found in 5 studies presenting zero prevalence of this morphology.

### 3.1. Global Tooth-Level Prevalence of Three-Rooted PMFM (Figure 6)

Sixty-eight studies estimated tooth-level prevalence of three-rooted PMFM. The total number of teeth in the meta-analysis was 37994, in which 5503 three-rooted PMFMs were found.

Global tooth-level prevalence of three-rooted PMFM was 8.85% (95%CI: 6.60%−11.39%) (*Q* = 4706.52, *p*=0.001, and *I*2 = 99%) by means of a random effect model. The occurrence ranged from 0% to 29% across the world.

By the subgroup analysis in accordance with the geographical locations, East Asian population revealed 24.1% (95% CI: 23%–25.2%) (range 15%–29%) (*Q* = 70.38, *p*=0.001, and *I*2 = 67%), Southeast Asia 13% (95% CI: 9%–17.7%) (range 8%–22%) (*Q* = 22.34, *p*=0.001, and *I*2 = 82%), South Asia 4.7% (95% CI: 2.5%–7.6%) (range 1%–9%) (*Q* = 28.16, *p*=0.001, and *I*2 = 86%), West Asia 4.4% (95% CI: 3.1%–5.8%) (range 2%–13%) (*Q* = 55.969, *p*=0.001, and *I*2 = 80%), Europe 2% (95% CI: 1.3%–2.9%) (range 0%–8%) (*Q* = 64.246, *p*=0.001, and *I*2 = 78%), America 1.8% (95% CI: 0.2%–4.6%) (range 0%–6%) (*Q* = 74.096, *p*=0.001, and *I*2 = 92%), and Africa 0.9% (95%CI: 0.3%–1.9%) (range 0.5%–1.1%) (*Q* = 0.451, *p* = 0.5, and *I*2 = 0%).

### 3.2. Global Patient-Level Prevalence of Three-Rooted PMFM (Figure 7)

Forty-four studies reported patient-level prevalence of three-rooted PMFM. The total number of patients in the meta-analysis was 16836, of which 2535 patients had three-rooted PMFM.

Global patient-level prevalence of three-rooted PMFM was 10.3% (95%CI: 6.9%–14.4%) ( *Q* = 2874.974, *p*=0.001, and *I*2 = 98%) by random effect model. The prevalence ranged between 0% and 35% around the world.

The patient-level prevalence of three-rooted PMFM was then presented by the subgroup analysis with respect to the different geographical regions. In such case, East Asian population revealed 28.8% (95%CI: 27.3%–30.4%) (range 22%–35%) (*Q* = 37.983, *p*=0.001, and *I*2 = 55%), South Asia 5.6% (95%CI: 2.2%–10.2%) (range 1%–11%) (*Q* = 21.774, *p*=0.001, and *I*2 = 86%), West Asia 4.4% (95%CI: 2.9%–6.1%) (range 2%–13%) (*Q* = 56.114, *p*=0.001, and *I*2 = 84%), America 2.1% (95%CI: 0.0%–5.9%) (range 0%–9%) (*Q* = 50.82, *p*=0.001, and *I*2 = 92%), and Europe 1% (95%CI: 0.1%–2.4%) (range 0%–4%) (*Q* = 42.03, *p*=0.001, and *I*2 = 86%).

There was not enough data to calculate the combined estimates for both Southeast Asia and Africa.

### 3.3. Global Patient-Level Prevalence of Bilateral Symmetry of Three-Rooted PMFM (Figure 8)

The data from 40 studies allowed us to calculate the patient-level prevalence of bilateral symmetrical distribution of three-rooted PMFM. A total of 2326 patients revealed three-rooted PMFMs. Of these, 1311 had three-rooted PMFMs on both sides of the mandible.

Global patient-level prevalence of bilateral symmetry of three-rooted PMFM was 46.22% (95%CI: 39.13%–53.39%) (*Q* = 385.13, *p*=0.001, and *I*2 = 90%) by random effect model. The prevalence ranged from 0% to 100%.

The patient-level prevalence of bilateral symmetry of three-rooted PMFMs was then explored by the subgroup analysis with regard to the different geographical locations. In this scenario, East Asian population revealed 60.3% (95%CI: 56.3%–64.2%) (range 49%–79%) (*Q* = 41.478, *p*=0.001, and *I*2 = 64%), South Asia 38.6% (95%CI: 0.0%–91.7%) (range 0%–70%) (*Q* = 108.934, *p*=0.001, and *I*2 = 97%), West Asia 37.5% (95%CI: 23.4%–52.7%) (range 0%–100%) (*Q* = 27.498, *p*=0.001, and *I*2 = 67%), America 35.1% (95%CI: 17.9%–54.4%) (range 33%–35%) (*Q* = 0.004, *p* = 0.95, and *I*2 = 0%), and Europe 18.7% (95%CI: 3%–41.5%) (range 0%–60%) (*Q* = 19.515, *p*=0.001, and *I*2 = 74%).

There was not enough information to calculate pooled estimates for Southeast Asia and Africa.

### 3.4. Tooth-Level Prevalence of Three-Rooted PMFM according to Sides of the Mandible (Subgroup Analysis)

Thirty-five studies allowed us to calculate the tooth-level distribution of three-rooted PMFM according to sides of the mandible. On the right side, the total number of teeth was 12604, of which 2631 were three-rooted. On the left side, the total number of teeth was 12483, of which 2043 were three-rooted.

The global prevalence of three-rooted PMFM on the right side of the mandible was 16% (95%CI: 12.2%–20.2%) (*Q* = 1261.665, *p*=0.001, and *I*2 = 97%), ranging between 1% and 34%. On the other hand, the global prevalence of three-rooted PMFM on the left side of the mandible was 12.1% (95%CI: 9.2%–15.4%) (*Q* = 950.242, *p*=0.001, and *I*2 = 96%), ranging from 0% to 31%. This signified the right-side predominance of the three-rooted PMFM.

### 3.5. Tooth-Level Prevalence of Three-Rooted PMFM according to Gender (Subgroup Analysis)

Thirty-five studies permitted us to estimate the tooth-level distribution of three-rooted PMFM according to gender. The total number of male PMFMs was 12922, of which 2393 had three roots. The total number of female PMFMs was 13313, of which 2190 were three-rooted.

The global prevalence of three-rooted PMFMs in male patients was 13.3% (95%CI: 9.8%–17.3%) (*Q* = 1348.191, *p*=0.001, and *I*2 = 97%), ranging between 1% and 32%. On the other hand, the global prevalence of three-rooted PMFMs in female patients was 13% (95%CI: 9.7%–16.7%) (*Q* = 1219.977, *p*=0.001, and *I*2 = 97%), ranging from 0% to 30%. This pointed out that there was no feature of sexual dimorphism in this case.

### 3.6. Global Tooth-Level Prevalence of Radix Entomolaris and Radix Paramolaris (Sensitivity Analysis)

To undertake sensitivity analysis, we excluded the studies which did not use the terms “RE” and “RP.” We included the studies that used the terms “RE” and “RP” in the sensitivity analysis.

Forty-three studies allowed us to estimate the tooth-level prevalence of Radix Entomolaris (RE) and Radix Paramolaris (RP). The total number of teeth was 28822. Of these, RE comprised 5056 and RP 21.

Global tooth-level prevalence of RE and RP was 12.3% (95%CI: 9.3%–15.7%) (*Q* = 2929.107, *p*=0.001, and *I*2 = 99%), ranging from 0% to 29% and 0.1% (95%CI: 0.0%–0.1%) (*Q* = 61.672, *p* = 0.03, and *I*2 = 30%), running between 0% and 2%, respectively.

### 3.7. Publication Bias Test

Publication bias test revealed that funnel plot asymmetry was seen in Ι global tooth-level prevalence of three-rooted PMFM (Figure 9) and ΙΙΙ global patient-level prevalence of bilateral symmetry of three-rooted PMFM (Figure 10). Doi plots also showed “major asymmetry” for both results. LFK indexes were −4.02 for objective Ι and −3.19 for objective ΙΙΙ.

Symmetrical funnel plot resulted from ΙΙ global patient-level prevalence of three-rooted PMFM (Figure 11). Doi plot also pointed out “no asymmetry.” LFK index was 0.05 for ΙΙ.

1944 of records were identified through PubMed, Goggle Scholar, Research Gate, ProQuest, and LILACS database search. 1711 duplicates and irrelevant records were excluded. 233 of records were screened. 108 full-text articles were assessed for eligibility.

Thirty-six full-text articles were excluded: (1) 6 studies that did not report the age of the patients and developmental condition of root apex, (2) 5 studies of which primary objectives were MMC, MR, and IM of the teeth, (3) 9 studies that used the methods, not being CBCT, (4) 3 in vitro studies that used CBCT, (5) 3 studies that did not report research methods and settings, (6) 1 study that used the combined method (PR + Spiral CT), (7) 1 study from which the data cannot be extracted, (8) 2 studies in which 5- and 6-year-old children were sampled, (9) 4 studies whose data were overlapped, (10) 1 study of contemporary cadaver sample, and (11) 1 thesis which was later published.

125 of records were excluded due to the following: (1) 25 in vitro studies, (2) 62 case reports, (3) 16 systematic reviews, (4) 10 that used X-ray methods, (5) 1 of extraction methods, (6) 1 of treatment modalities, (7) 1 book chapter, (8) 1 that combined clinical investigation and Spiral CT, (9) 1 editorial, and (10) 7 Chinese language studies of which full-texts were not available to access.

Seventy-two studies were included in both qualitative and quantitative analyses.

## 4. Discussion

### 4.1. Summary of Main Findings

Global tooth-level prevalence of three-rooted PMFM was 9% ranging from 0% in Chilean, Italian White, Brazilian, Serbian, and Russian populations [30, 75, 76, 78, 85] to 29% in the Chinese populations [47, 93]. By the subgroup analysis of objective Ι with regard to the different geographical locations, East Asia, Southeast Asia, South Asia, West Asia, Europe, America, and Africa demonstrated 24%, 13%, 5%, more than 4%, 2%, 1.8%, and nearly 1%, respectively. The proportions were apparently downgraded from the East across Asia to the West. We believe that globalization, migration, and blended ethnicity may influence the prevalence of three-rooted PMFM, especially in the Native American population.

Global patient-level prevalence of three-rooted PMFM was 10% ranging from 0% in Chilean, Italian White, Brazilian, Serbian, and Russian populations [30, 75, 76, 78, 85] to 35% in a Chinese population [47]. By the subgroup analysis of objective ΙΙ according to the different geographical regions, East Asia, South Asia, West Asia, America, and Europe comprised 29%, 6%, more than 4%, over 2%, and 1% individually. Tooth-level data was usually lower in proportion than patient-level data, significantly in East Asia. It seems to be basically originated from which the number of teeth, if being bilaterally present, is more numerous than the number of patients to whom the teeth belong. This could affect the denominator of the pooled estimate. The higher the denominator count, the lower the resulting proportion.

Global patient-level bilateral symmetry of three-rooted PMFM was 46% ranging from 0% in Iranian, Turkish, Pakistani, and Egyptian populations [35, 41, 55, 66, 83] to 100% in an Iranian population [40]. By the subgroup analysis of objective ΙΙΙ across the different geographical locations, East Asia, South Asia, West Asia, America, and Europe displayed 60%, nearly 39%, approximately 38%, 35%, and over 18%, respectively. Although a Middle East country was ranking the highest of the range of bilateral symmetry, the East Asia group pooled up to 60% at this parameter.

For the objective ΙΙΙ, a wide range of the prevalence and broad confidence interval of each subset were investigated, especially in South Asia and West Asia. This reflects the small sample size of each subgroup in these areas.

#### 4.1.1. Subgroup Analyses

Global tooth-level prevalence of right three-rooted PMFM was 16%, ranging from 1% in Egypt population [83] and then 2% in a Brazilian population [36] up to 34% in the Han population of China [47]. Global tooth-level prevalence of left three-rooted PMFM was 12% fluctuating between 0% in both Egypt [83] and an Iranian population [35] and 31% in a Western Chinese population [89]. We identify that the prevalence of three-rooted PMFM could be seen in nearly one-third of the number of teeth in East Asia. In this meta-analysis, the prevalence of three-rooted PMFM was definitely skewed towards the right side of the mandible.

Global tooth-level prevalence of three-rooted PMFM in male patients was more than 13% ranging from 1% in both Brazilian [36] and Egyptian [83] populations to 32% in Han people of China [47]. Global tooth-level prevalence of three-rooted PMFM in female patients was 13% ranging from 0% in an Egypt population [83] and then 2% in Brazilian [36], Turkish [41], and Israeli [2] populations up to 30% in a Western Chinese population [89]. As a result, the occurrence of three-rooted PMFM was not sexually dimorphic in our meta-analysis.

#### 4.1.2. Sensitivity Analysis

Global tooth-level prevalence of Radix Entomolaris was more than 12% limiting between 0% in the Egyptian population [83] and 29% in the Han and Chengdu populations of China [47, 93]. Global tooth-level prevalence of Radix Paramolaris was 0.1% ranging from 0% in nearly two-thirds of the meta-analyzed studies, then around 1% in Indian [44], Greece [3], and Israeli [2] populations, respectively, and nearly 2% in Spanish [73] population. We postulate that RP could be less reported and underestimated due to its lesser clinical significance than RE.

### 4.2. Comparison with the Findings of Previous Reviews

There were an increasing number of pieces of evidence, particularly systematic reviews and meta-analyses in the subject of endodontic anatomy.

One earliest systematic review of the scope investigated that the prevalence of the third root in PMFMs across the world demonstrated 13% [24]. Our meta-analysis investigated a 4% reduction in the pooled tooth-level prevalence compared to the previous one. On the other hand, the prevalence of RE found in our analysis was comparable to the result of this former investigation. We estimate that blended ethnicities resulting from globalization may have an impact on the prevalence of the third root in the Western world. Interestingly, 2 studies and 1 case report using the CBCT method were included in this former evidence [24]. Specifically, the prevalence of three-rooted PMFM in a dental school sample of Germany and an English Caucasian population demonstrated 0.7% and 3.3%, resulting from the narrative synthesis in this previous review [18, 94]. The pooled patient-level prevalence in our present review displayed 1% ranging from 0% to 4% in Europe. So, the two reviews were in agreement at this point. Additionally, both of the European [18, 94] studies included in this review reported no bilateral symmetry of this morphology. Our meta-analysis pointed out that the pooled bilateral symmetry of three-rooted PMFM in Europe was the lowest out of all the continents.

Then, a systematic review of Brazil investigated the prevalence of three-rooted PMFMs revealed between 1.5% and 3% in such population [25]. The range in our present meta-analysis comprised from 0% to 6% in the American continents, including Brazil. Zero-prevalence studies [30], some studies' samples restricted to the White population [43], and blended ethnicities including Asian-American [46] used in some studies may broaden the range. Surprisingly, one of the earliest investigations detected 22% of three-rooted PMFMs found in the Canadian Eskimo sample [95]. So, we conclude that the anatomy of migrants could differ from that of the original natives in the continents.

Tomaszewska and coauthors found that the pooled prevalence of three-rooted PMFMs comprised 8.9% [27] after the meta-analysis of 35 chosen studies. This finding was nearly similar to the global tooth-level prevalence of our analysis. However, the former meta-analysis did not further investigate the patient-level prevalence and bilateral symmetry of three-rooted PMFMs, subgroup, and sensitivity analyses to explore heterogeneity. Additionally, the investigators used studies of different research methodologies to pool the estimate.

Consequently, a recent systematic review of Saudi Arabia qualitatively observed that the occurrence of RE ranged from 2.9% to 6.07% [26]. The range was slightly wider than our review's findings. In our review, the prevalence of RE in West Asia, including Saudi Arabia, featured from 2% in Israeli [2] to 5% in the Iranian population [40]. We postulate that the European descend Israeli population may be implicated to become lower prevalence. We also suspect that the previous review was limited to the Saudi population, not all West Asia area, and showed a methodological discrepancy in comparison with our review. The primary studies in the Saudi review [26] used the X-ray method and the combined methodology of CBCT and X-ray. However, 60% of RE was limitedly investigated in periapical X-rays [11]. The straight distolingual root could be overlapped by the distobuccal root. As a result, the X-ray method may lead to a deficiency in the estimation of RE.

In addition, the tooth-level prevalence of three-rooted PMFM was more numerous in females than in males in the review of Saudi people [26]. In our findings, only the Nepalese population [38] also showed female determinants of three-rooted PMFMs clearly. Alternatively, the various East Asia studies [47, 50, 56, 77] demonstrated that three-rooted PMFMs were more often seen in males than females.

However, distolingual root or two distal roots could not be strictly defined as “RE.” Centrolingual and distobuccal positions of the extra root may also be found (Figures 1(b) and 1(e)) [2–4]. All RE can be three-rooted. However, every three-rooted PMFMs could not be stated as “RE.” So, we contributed and categorized the variable positions of the third root, with reference to the latest available evidence (Figure 1) [2–4].

Importantly, patient-level prevalence and bilateral symmetry of three-rooted PMFMs were missed to report in the previous reviews. They also did not undergo the proportional meta-analysis for Southeast Asia and South Asia by subgroup analysis. They should report RP as a separate entity. All these variables should also be considered as clinically significant factors.

### 4.3. Significant Findings apart from the Previous Reviews

In addition to objectives ΙΙ and ΙΙΙ (patient-level prevalence), subgroup analyses of Southeast Asia and South Asia were significant aside from the previous observation.

In Southeast Asia, the pooled tooth-level prevalence of three-rooted PMFM was 13%, ranging between 8% and 22% in this meta-analysis. The lowest 8% was found in the study, which restricted the sample solely to include Malay ethnicity [67]. The highest 22% was seen in the study, in which more than 90% of the sample was Chinese [70]. Both studies were conducted in Malaysia. So, the prevalence of three-rooted PMFM depended upon the demographic characteristic of the participants even in the same country. The prevalence of three-rooted PMFMs was 10% in Burmese [96] and 19% in Thai populations [97] regardless of considering different research methods used. As a result, we conclude that the values of the previous studies were within the range of our meta-analysis.

In South Asia, the summarized tooth-level prevalence of three-rooted PMFM was 4.7%, fluctuating between 1% in Karachi people of Pakistan [66] and 9% in Nepal [38] by the subgroup analysis of our review. However, the Nepal sample was a mixture of Aryan, speaking the Indo-European language, and Mongoloid people. Chandra and coworkers [19] found that the incidence of RE was 13% in tooth-level and more than 18% in patient-level of an Indian population, being obviously outlying when compared with our investigation. The patient-level prevalence of three-rooted PMFMs in our meta-analysis indicated a 6% pooled estimate in South Asia and almost 11% in the Nepalese population [38] at the upper boundary. At this point, we suggest that ethnical diversity, sample size, and the number of selected studies could be the impact factors on pooling the combined approximation.

As a result, the Mongoloid descents were positively associated with the prevalence of three-rooted PMFMs in both Southeast Asia and South Asia regions.

Additionally, “RP” Radix Paramolaris is less clinically significant than “RE,” although having both anatomical and anthropological significance. Unfortunately, the previous reviews did not point out the description of RP, although the pooled prevalence of RP in our meta-analysis demonstrated 0.1%. RP was mostly found in nearly 2% of Spanish [73] and Greece populations of Europe [3], Jews who could be European or Arab descendants [2], and Indians [44] in our present meta-analysis. Less than 0.5% of RP was found in some Saudi [31] and Turkish [55] populations. Interestingly, RP was not reported in East Asian and African studies. Additionally, Radix Paramolaris of the mesial root of PMFM [2] (Figure 1(f)) could not be detected prominently in periapical X-ray.

### 4.4. Strengths and Weaknesses of the Meta-Analysis

In contrast to the previous systematic reviews, patient-level prevalence and bilateral symmetry of three-rooted PMFMs were the most striking features of the review. Additionally, the Asia continent was subdivided into four geographical locations to transparent different levels of prevalence of three-rooted PMFMs in the continent. RP was also reported as a pooled prevalence, which could be anthropologically significant.

Literature search was thoroughly done across five databases for the review (Figure 4). To overcome language bias, 4 studies [33–35, 47] were translated from native languages to English (Table 1). We emphasize that findings could be more generalizable to the global population when foreign language studies were included as much as possible.

Being in vivo study was one eligible criterion for our meta-analysis. In vitro needs extraction and can lead to underestimation of the findings due to root fracture during the surgical procedure. Validity of measurement in the included studies could be properly achieved by the CBCT method. Counting the number of roots could be objectively done by viewing CBCT images, especially in axial slices. Thereby, measurement bias could be reduced.

As a weakness, there was no adequate raw data for the patient-level prevalence of Africa and Southeast Asia. And zero-prevalence studies were included in the meta-analysis (Table 1). This may skew the pooled findings to be underestimated or overestimated. To explore heterogeneity, subgroup analysis was not done through different age groups. However, the presence of the third root in PMFM could not be influenced by the age of the population. Additionally, the prevalence of three-rooted PMFMs, with regard to the different ethnicities across the world, was not explored in this meta-analysis. Blended ethnicities, migration, and globalization may not guarantee the representativeness of each ethnicity.

In Africa, the pooled estimate of our meta-analysis revealed 0.9% ranging from 0.5% in Egypt [83] to 1.1% in South Africa [87]. This finding was slightly inferior to the previous report demonstrating the prevalence of three-rooted PMFMs more than 3% in the Senegalese population [98]. Otherwise, it reached nearly an agreement with the former one reporting 0.65% of three-rooted PMFM in Egyptian residents of Saudi Arabia [99]. However, we conclude that only two studies selected for Africa in our analysis may not be generalizable to the whole continent.

Similarly, there was no eligible study for the Australian continent. More than three-quarters of the Australians were of European descent, and the remaining were a mixture of different ethnicities [100]. As a result, we postulate that the prevalence of three-rooted PMFM in Australians may not be seen different from that of the Western world. Consequently, a recent worldwide cross-sectional study [101] with a meta-analysis found that the prevalence of two distal roots in PMFM comprised 4% of the White Australian population. This finding was slightly superior to the upper bound of the prevalence of three-rooted PMFM in Europe of our meta-analysis. As a result, this finding from 214 White patients of Melbourne and a single-center study [101] could not be generalizable to the whole population of the Australian continent. Additionally, the aboriginal population of Australia should not be neglected to consider.

Consequently, the internal morphology of the third root of PMFM was not included in the objectives of this meta-analysis. More precisely, the importance of the radix molar and its associated clinical features should also be conveyed to clinicians.

### 4.5. Limitations

Slightly more than half of the included studies were categorized as moderate risk of bias, whereas another half had a high risk of bias. Bias may arise from the weaknesses of research methodology in some selected studies (Figure 5).

More than 80% of the studies did not formally set a sampling frame. Strictly, 4% of the eligible studies conducted random sequence generation. Only 15% of the included studies used the calculated sample size. Up to 95% of the selected picked up the data from the single centers of the city or the province. As a result, selection bias, over coverage or under coverage on the population of interest, and deficient in generalization [102] may arise and manifest on the findings of the meta-analysis.

Nearly 50% of the selected studies undertook a reliability test on measurement. And only 33% of these reported the outcomes thoroughly. Consequently, reporting bias and performance bias [102] may partly jeopardize the results of this review.

In addition, 19 studies of the review presented the primary objective, which matched the primary outcome of the review. Of these, 12 studies thoroughly reported the variables and allowed us to calculate the pooled estimates. So, incomplete outcome reporting may be investigated even in the studies in which the main objective was to assess the prevalence of three-rooted PMFM or RE.

Consequently, publication biases were investigated in cases of global tooth-level prevalence and global patient-level bilateral symmetry of three-rooted PMFMs.

In Figure 9, the small studies crowded at the funnel base. And they represented extreme values (proportion/double arcsine prevalence) at the *x*-axis. This means that these small studies exhibited a large effect. Also, in Figure 10, there was sparse or lack of studies at the left-hand base of the funnel plot. This means asymmetry. The asymmetrical plot and the small studies having great effect indicated publication bias.

The studies, scattering equal over both sides of the funnel plot, were detected in the case of the global patient-level prevalence of three-rooted PMFMs (Figure 11), showing “no asymmetry.”

All of the publication bias tests were additionally confirmed by Doi plots and LFK indexes.

### 4.6. Heterogeneity

Q statistics mean the difference in the prevalence of three-rooted PMFMs of individual studies included in the meta-analyses. *I*2 statistics mean the percentage of variation in the prevalence of three-rooted PMFMs of such studies. An increase in the two values indicates the progress of heterogeneity.

**Figure 6:**
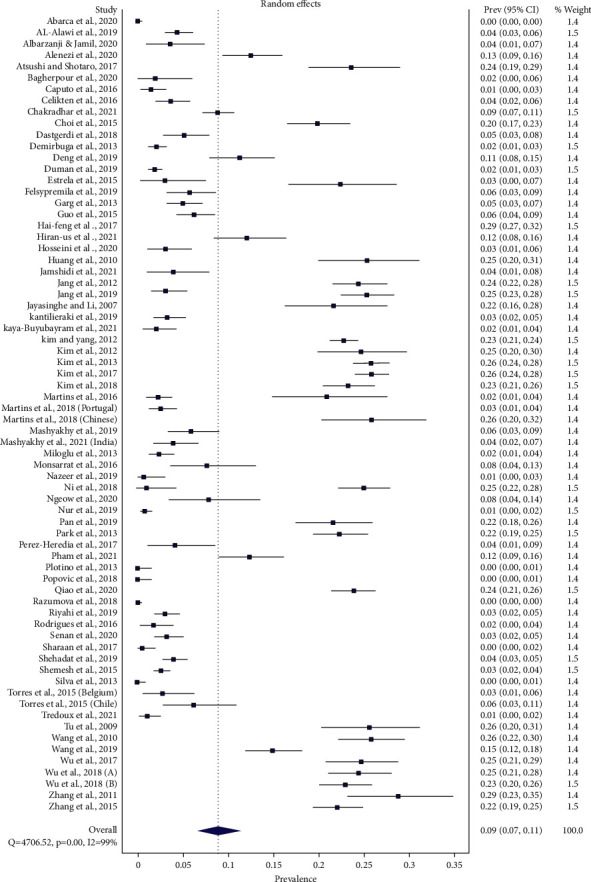
Global tooth-level prevalence of three-rooted PMFM.

From the global tooth-level prevalence of three-rooted PMFMs (objective Ι), *I*2 demonstrated considerable heterogeneity (Figure 6). To explore heterogeneity, subgroup analysis according to the different geographical locations was undertaken. After the analysis, *I*2 was downgraded from the combined estimate of 99% to 0% in Africa at the upper bound and to 92% in America at the lower.

**Figure 7:**
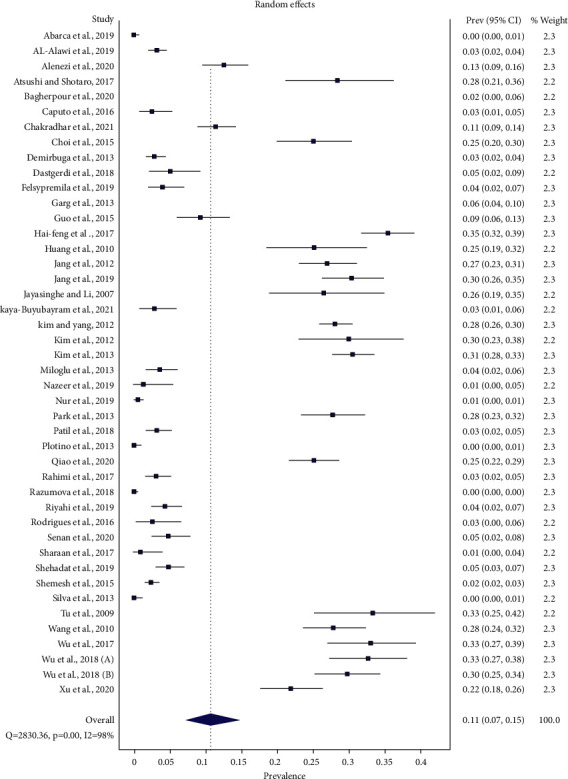
Global patient-level prevalence of three-rooted PMFM.

From the global patient-level prevalence of three-rooted PMFMs (objective ΙΙ), *I*2 dropped from the pooled estimate of 98% (Figure 7) to 55% in East Asia and to 92% in America, investigated by the subgroup analysis.

**Figure 8:**
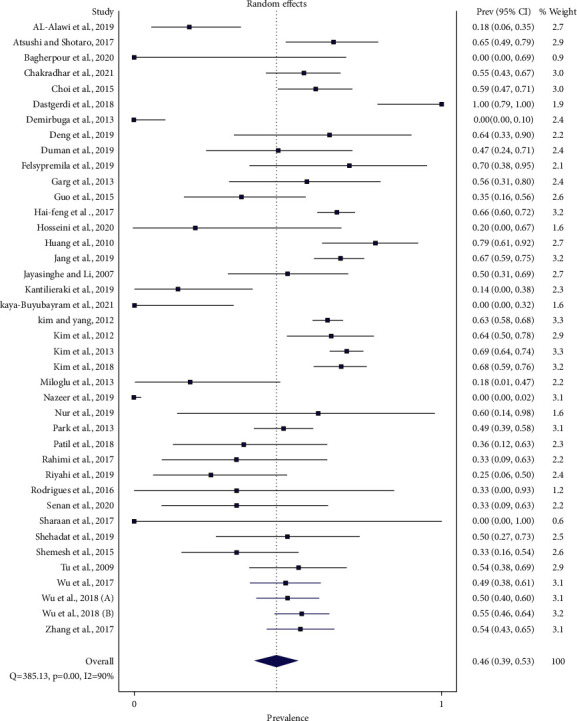
Global patient-level bilateral symmetry of three-rooted PMFM.

**Figure 9:**
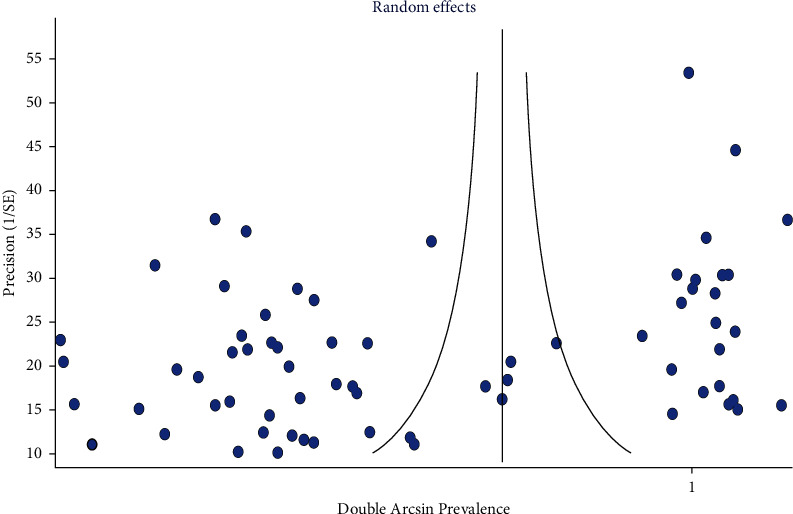
Funnel plot of global tooth-level prevalence of three-rooted PMFM (Ι).

**Figure 10:**
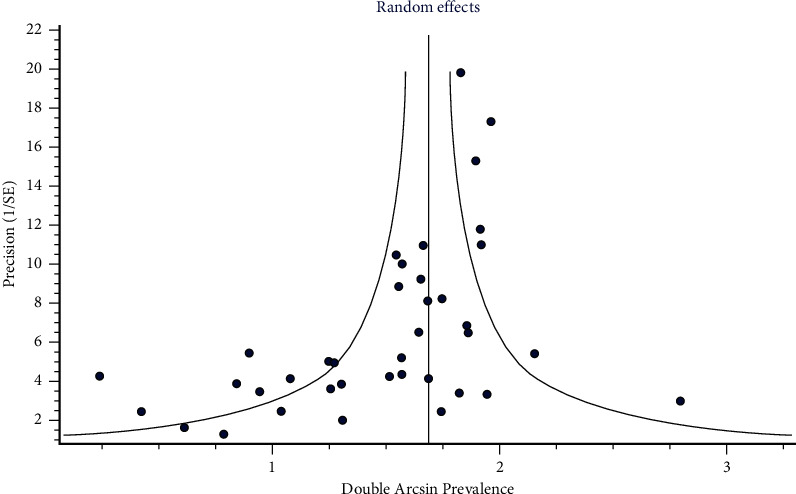
Funnel plot of global patient-level prevalence of bilateral symmetrical distribution of three-rooted PMFM.

**Figure 11:**
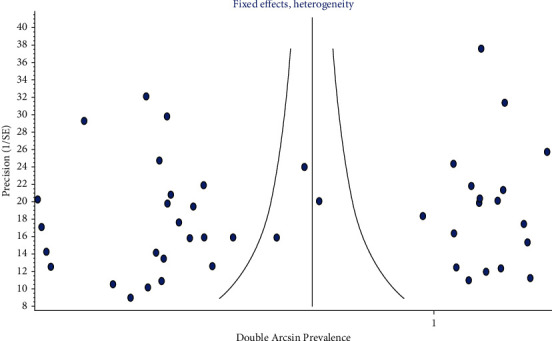
Funnel plot of global patient-level prevalence of three-rooted PMFM.

From the global patient-level prevalence of bilateral symmetry of three-rooted PMFMs (objective ΙΙΙ), *I*2 rose from 90% (Figure 8) to 97% in South Asia and descended to 0% in America after the subgroup analysis.

As a result, *I*2 declined at least 6% and as much as 99% after the subgroup analyses.

So, the heterogeneity mainly emerged from the different geographical locations. The rest of the heterogeneity was rooted partially from clinical, statistical, and methodological factors.

Most designs of the chosen studies were descriptive and retrospective in character. 19% of the selected studies were analytical designs (Table 1). Only 1 study approached prospective [38]. These contexts figured out the clinical and methodological heterogeneities. Blending of Asians and Caucasian samples [46] and limitation to select Caucasians [81] may lead to over and under estimation of the prevalence.

The sensitivity analysis drove the heterogeneity of the overall estimate of 99% to 30% in the event of the global prevalence of Radix Paramolaris (RP). It demonstrates that the prevalence around the world displayed agreement among the chosen studies in such a point.

There was no significant variable heterogeneity in the right and left and gender distributions compared with the pooled tooth-level estimate.

### 4.7. Direction of Future Studies

We have a plan to intervene in a future systematic review and meta-analysis to find the clinical factors connected with three-rooted PMFMs across the global population. The present meta-analysis should be updated to additionally assess variations in the position of the third root along the circumference of PMFMs in the distance future.

### 4.8. Take-Home Messages for Clinicians

Dental practitioners should generally keep in mind, according to the key estimates of the present meta-analysis, the following:

- 9% of PMFMs in the world population demonstrated 3 rooted PMFMs
- 10% of the people across the world had three-rooted PMFMs
- 45% of the people who had three-rooted PMFMs revealed a bilateral symmetrical distribution of such morphology
- The global prevalence of three-rooted PMFM was not identified as a sex determinant but showed right-side predominance
- The global tooth-level prevalence of Radix Entomolaris and Radix Paramolaris was 12% and 0.1%, respectively

## 5. Conclusion

To the best of our knowledge, the prevalence of three-rooted PMFMs was concerned with different geographical locations all over the world and by the widespread habitation of Mongoloid descents. We suggest that globalization, blending, and interaction among ethnicities may have a huge impact on the reduction or accentuation of the anatomical significance in some populations.

## Figures and Tables

**Table 1:** Population, country, number of patients, number of teeth, geographic location, condition, setting, and study design of the included studies.

No.	Study name	Population	Country	Number of patients	Number of teeth (PMFM)	Geographic location	Condition termed as	CBCT (setting)	Study design
1	Abarca et al. ^*∗*^ [30]	Chilean population	Chile	289	510	America	“Number of roots”	Gendex® GXCB-500, 120 kVp	Cross-sectional, descriptive, and retrospective
								5 mA	
								0.2 mm voxel size	
2	Al-Alawi et al. [31]	Saudi Arabian population	Saudi Arabia	450	741	West Asia	“Radix Molaris”	(i) ProMax 3D Max (90 kVp, 10 mA)	Cross-sectional, descriptive, and retrospective
								(ii) Galileos Comfort (85 kVp, 7 mA, and voxel size 0.2–0.4 mm)	
								(iii) CS9300 (90 kVp, 10–15 mA, and voxel size 0.9 mm)	
3	Albarzanji and Jamil [32]	Iraqi population	Iraq	100	141	West Asia	“Extra distal root”	NewTom VGi 9000	Cross-sectional, descriptive, and retrospective
								110 kVp	
								19 mA	
								015 mm voxel size	
								FOV 12 × 7.5 cm	
4	Alenezi et al. (*τ*) [33]	Saudi Arabian population	Saudi Arabia	400	400	West Asia	“Three-rooted”	ProMax 3D Max	Cross-sectional, descriptive, and retrospective
								Voxel size 0.2–0.6 mm	
5	Atsushi and Shotaro (*τ*) [34]	Japanese population	Japan	141	279	East Asia	“Distolingual root”	ProMax 3D	Cross-sectional, descriptive, and retrospective
								84 kVp	
								12 mA	
								FOV 0.16 × 0.16 × 0.16 mm	
6	Bagherpour et al. (*τ*) [35]	Iranian population	Iran	100	100	West Asia	“Three roots”	ProMax 3D Planmeca	Cross-sectional, descriptive, and retrospective
								Not reported detail setting	
7	Caputo et al. [36]	Brazilian population	Brazil	198	342	America	“Supernumerary root”	Gendex CB 500	Cross-sectional, descriptive, and retrospective
								Voxel size 0.2 mm	
								FOV 8 × 14 cm	
8	Celikten et al. [37]	Turkish Cypriot population	Cypriot	272	384	Europe	“Three roots”	NewTom 3G, FOV 9, 110 kVp, 0.3 mm voxel size	Cross-sectional, descriptive, and retrospective
9	Chakradhar et al. [38]	Nepalese population	Nepal	571	1142	South Asia	“Radix Entomolaris”	Rainbow TM CT	Cross-sectional, analytical, and prospective
								100 kVp	
								12 mA	
								16 × 18 cm FOV	
								Voxel size 300 *μ*m	
10	Choi et al. [39]	South Korean population	South Korea	264	528	East Asia	“Distolingual root”	Alphard Vega scanners	Cross-sectional, descriptive, and retrospective
								FOV 512 × 512 mm	
								80 kVp	
								5 mA	
								Voxel size of 0.39 mm	
11	Dastgerdi et al. [40]	Iranian population	Iran	156	312	West Asia	“Distolingual root”	NewTom	Cross-sectional, descriptive, and retrospective
								FOV 8 × 5 cm	
								mA 12	
								kVp 85	
								Voxel size 0.3 mm	
12	Demirbuga et al. [41]	Turkish population from Cappadocia	Turkey	605	823	Europe	“Third root”	NewTom 5G	Cross-sectional, descriptive, and retrospective
								Not reported detail setting	
13	Deng et al. [42]	Malaysian population	Malaysia	301	301	Southeast Asia	“Number of roots”	3D Planmeca ProMax	Cross-sectional, descriptive, and retrospective
								FOV 80 × 80 mm	
								90 kVp	
								10 mA	
								Voxel size 0.32 mm	
14	Duman et al. [6]	Turkish population	Turkey	850	1318	Europe	“Radix Entomolaris”	NewTom 5G	Cross-sectional, descriptive, and retrospective
								Voxel size 0.125 mm	
15	Estrela et al. [43]	Brazilian population	Brazil	618	100	America	“Number of roots”	PreXion 3D	Cross-sectional, descriptive, and retrospective
								FOV 56.00 mm	
								90 kVp	
								4 mA	
								Voxel size of 0.1 mm	
16	Felsypremila et al. [44]	Indian subpopulation	India	246	299	South Asia	“Radix Entomolaris” and “Radix Paramolaris”	Kodak 9500 3D	Cross-sectional, descriptive, and retrospective
								FOV 18 × 20 mm	
								60–90 kVp	
								2–15 mA	
17	Garg et al. ($) [45]	Indian population	India	250	500	South Asia	“Distolingual root”	SCT Samatum Balance	Cross-sectional, descriptive, and retrospective
								130 kVp	
								90–135 mA	
18	Guo et al. [46]	American population	United States	248	496	America	“Distolingual root”	Sirona dental system	Cross-sectional, descriptive, and retrospective
								Voxel size 0.3 mm^3^	
								FOV 15 × 15 × 15 cm^3^	
								85 kVp	
								5–7 mA	
19	Hai-feng et al. (*τ*) [47]	Chinese population	China	656	1312	East Asia	“Extra distal root”	Planmeca Romexis	Cross-sectional, descriptive, and retrospective
								90 kVp	
								14 mA	
								0.2 mm voxel size	
								FOV 40 × 40 mm	
20	Hiran-us et al. [48]	Thailand population	Thailand	248	256	Southeast Asia	“Three-rooted”	3D Accuitomo	Cross-sectional, descriptive, and retrospective
								FOV 4 × 4 cm/17 × 12 cm	
								90 kVp	
								5.8 mA	
								Voxel size of 0.08–0.25 mm^3^	
21	Hosseini et al. [49]	Iranian population	Iran	113	200	West Asia	“Radix Entomolaris”	NewTom 5G	Cross-sectional, descriptive, and retrospective
								110 kVp	
								10 mA	
								75 *μ*m voxel size	
22	Huang et al. [50]	Taiwanese population	Taiwan	151	237	East Asia	“Three roots”	i-CAT	Cross-sectional, descriptive, and retrospective
								120 kVp	
								5 mA	
23	Jamshidi et al. [51]	Iranian population	Iran	500	129	West Asia	“Three roots”	ProMax 3D	Cross-sectional, descriptive, and analytic
								84 kVp	
								10 mA	
24	Jang et al. [52]	South Korean population	South Korea	472	780	East Asia	“Three-rooted” and “distolingual root”	Implagraphy	Cross-sectional and descriptive
								Voxel size of 200 *μ*m	
								FOV 144 × 121	
25	Jang et al. [53]	South Korean population	South Korea	451	902	East Asia	“Distolingual root”	Dinnova system	Cross-sectional, analytic, and retrospective
								80 kVp	
								9.0 mA	
								FOV 10 × 10 cm	
								Voxel size of 0.167 mm^3^	
26	Jayasinghe and Li [54]	Hong Kong Chinese population	Hong Kong	117	203	East Asia	“Distolingual root”	Spiral CT	Cross-sectional, descriptive, and retrospective
								CT HiSpeed	
								150 mA	
								512 × 512 pixel	
27	Kantilieraki et al. [3]	Greek population	Greek	592	478	Europe	“Radix Entomolaris” and “Radix Paramolaris”	NewTom VGi Evo	Cross-sectional, descriptive, and retrospective
								110 kVp	
								3 mA	
								Scanora® 3D	
								90 kVp	
								13 mA	
								Voxel 0.1 × 0.1 ×0.1 mm^3^	
28	Kaya-Buyukbayram et al. [55]	Turkish population	Turkey	177	242	Europe	“Radix Entomolaris” and “Radix Paramolaris”	3D Accuitomo 170	Cross-sectional, descriptive, and retrospective
								90 kVp	
								5 mA	
								250 *μ*m voxel size	
								FOV 140 × 100	
29	Kim and Yang [56]	South Korean population	South Korea	1400	2800	East Asia	“Distolingual root”	i-CAT	Cross-sectional, descriptive, and retrospective
								129 kVp	
								47.74 mA	
30	Kim et al. [57]	South Korean population	South Korea	150	300	East Asia	“Distolingual root”	Master 3DS	Cross-sectional, analytical, and retrospective
								90 kVp	
								3–7 mA	
								Voxel size 0.2–0.4 mm	
31	Kim et al. [58]	South Korean population	South Korea	976	1952	East Asia	“Extra distal root”	Dinnova system	Cross-sectional, descriptive, and retrospective
								80 kVp	
								9.0 mA	
								FOV 10 cm	
								Voxel size 0.167 mm^3^	
32	Kim et al. [12]	South Korean population	South Korea	979	1958	East Asia	“2 distal roots”	Dinnova system	Cross-sectional, descriptive, analytical, and retrospective
								80 kVp	
								9.0 mA	
								FOV 10 cm	
								Voxel size 0.167 mm^3^	
33	Kim et al. [59]	South Korean population	South Korea	432	864	East Asia	“Distolingual root”	CB Mercury	Cross-sectional, descriptive, analytical, and retrospective
								120 kVp	
								15 mA	
								FOV 10 cm	
								Voxel size 0.2 mm	
34	Martins et al. [60]	Portugal's population	Portugal	646	450	Europe	“Number of roots”	3D Planmeca ProMax	Cross-sectional, descriptive, and retrospective
								Large FOV	
								80 kVp	
								15 mA	
								Voxel size 0.02 mm	
35	Martins et al. [61]	Portugal's population and Chinese population	Portugal and China	670 (Portugal)	466 (Portugal)	Europe and East Asia	“Number of roots” and “Radix Entomolaris”	3D Planmeca ProMax	Cross-sectional, analytical, and retrospective
								Kodak 9500	
				120 (Chinese)	220 (Chinese)			Full arch FOV	
								Voxel size 0.02 mm	
36	Mashyakhy et al. [62]	Saudi Arabian population	Saudi Arabia	208	274	West Asia	“Three roots”	3D Accuitomo 170	Cross-sectional, analytical, and retrospective
								FOV 170 × 140 mm	
								90 kVp	
								5.8 mA	
								Voxel size 0.25 mm	
37	Mashyakhy et al. [63]	Indian population	India	150	255	South Asia	“Three roots”	3D Accuitomo 170	Cross-sectional, analytical, and retrospective
								FOV 170 × 140 mm	
								90 kVp	
								5–8 mA	
								Voxel size 0.25 mm (Saudi Arabia)	
								Kodak 9500 3D	
								FOV 18 × 20 cm	
								60–90 kVp	
								2–15 mA	
38	Miloglu et al. [64]	Turkish population	Turkey	307	533	Europe	“Extra distolingual root”	NewTom FP QR-DVT	Cross-sectional, descriptive, and retrospective
								110 kVp	
								15 mA	
39	Monsarrat et al. [65]	France population	France	102	130	Europe	“3 roots”	CS 9500 3D	Cross-sectional, analytical, and retrospective
								90 kVp	
								10 mA	
								Voxel size 200 *μ*m	
								FOV 90 × 150 mm	
40	Nazeer and Khan [66]	Pakistani population	Pakistan	78	142	South Asia	“Two distal roots”	Sirona dental system	Cross-sectional, descriptive, and retrospective
								85 kVp	
								7 mA	
41	Ngeow et al. [67]	Malaysian population	Malaysia	61	115	Southeast Asia	“3-rooted”	i-CAT	Cross-sectional, descriptive, and retrospective
								120 kVp	
								3–7 mA	
								0.3 mm voxel size	
								FOV 60 × 13 cm	
42	Ni et al. [68]	Chinese population	China	646	900	East Asia	“Distolingual root”	J. Morita	Cross-sectional, descriptive, and retrospective
								80 kVp	
								5 mA	
								FOV 8 × 8 cm	
43	Nur et al. [69]	Turkish population	Turkey	850	966	Europe	“Number of roots”	I-CAT Vision TM	Cross-sectional, descriptive, and retrospective
								120 kVp	
								18.54 mA	
								0.3 mm voxel size	
44	Pan et al. [70]	Malaysian population	Malaysia	208	370	Southeast Asia	“Radix Entomolaris”	KaVo 3D eXam	Cross-sectional and descriptive
								120 kVp	
								5 mA	
								Voxel size 0.25	
45	Park et al. [71]	South Korean population	South Korea	430	726	East Asia	“DL root”	CBCT	Cross-sectional, descriptive, and retrospective
								Not reported detail setting	
46	Patil et al. [72]	Saudi Arabian population	Saudi Arabia	428	Tooth-level data was not reported	Western Asia	“Three-rooted”	Scanora 3D	Cross-sectional, descriptive, and retrospective
								6 mA	
								89 kVp	
47	Perez-Heredia et al. [73]	Spanish population	Spain	112	121	Europe	“Third root”	9300 3D CBCT unit	Cross-sectional, descriptive, and retrospective
								90 kVp	
								4 mA	
								FOV 10 × 10 cm	
								Voxel size 0.18	
48	Pham and Le [74]	Vietnamese population	Vietnam	166	332	Southeast Asia	“Distolingual root”	Pica 330 Trio	Cross-sectional, descriptive, and retrospective
								FOV 8 5 cm	
49	Plotino et al. ^*∗*^ [75]	Italian White population	Italy	210	117	America	“Number of roots”	NewTom VGi vertical cone beam	Cross-sectional, descriptive, and prospective
								Not reported detail setting	
50	Popovic et al. ^*∗*^ [76]	Serbian population	Serbia	192	118	Europe	“Number of roots”	Orthophos XG 3D	Cross-sectional, descriptive, and retrospective
								Voxel size 160 m	
								Large FOV	
51	Qiao et al. [77]	Chinese population	China	587	1174	East Asia	“Radix Entomolaris”	3D Accuitomo	Cross-sectional, analytical, and retrospective
								85 kVp	
								4.5 mA	
								FOV 60 × 60 cm	
								Voxel size 0.125 mm	
52	Razumova et al. ^*∗*^ [78]	Moscow population	Russia	300	407	Europe	“Number of roots”	3D Exam®	Cross-sectional, descriptive, and retrospective
								FOV 23 17 cm	
								Voxel size 0.3	
								110 kVp	
53	Riyahi et al. [79]	Saudi Arabian population	Saudi Arabia	379	655	West Asia	“Three-rooted”	ProMax 3D	Cross-sectional, descriptive, and retrospective
								Voxel size 0.2–0.6 mm	
54	Rahimi et al. [80]	Iranian population	Iran	386	Tooth-level data was not reported	West Asia	“Radix Entomolaris”	NewTom VG 9000	Cross-sectional, descriptive, and retrospective
								120 kVp	
								150 mA	
55	Rodrigues et al. [81]	Brazilian population	Brazil	116	232	America	“Radix Entomolaris”	i-CAT	Cross-sectional, descriptive, and retrospective
								120 kVp	
								8 mA	
								0.25 mm voxel size	
56	Senan et al. [82]	Yemini population	Yemen	250	500	West Asia	“Radix Entomolaris”	Pax-Flex 3D	Cross-sectional, descriptive, and retrospective
								50–90 kVp	
								2–10 Ma	
								FOV 50 × 50 mm	
								Voxel size 120 *μ*m	
57	Sharaan and Elrawdy [83]	Egyptian population	Egypt	109	218	Africa	“Radix Entomolaris”	Scanora 3D	Cross-sectional, descriptive, and retrospective
								Voxel size 133 *μ*m	
								10 mA	
								90 kVp	
								FOV 14 × 16.5 cm	
58	Shehadat et al. [84]	UAE population	UAE	475	807	West Asia	“Three roots”	Not reported detail	Cross-sectional, descriptive, and retrospective
59	Shemesh et al. [2]	Israeli population	Israel	1020	1229	West Asia	“Radix Entomolaris” and “Radix Paramolaris”	ASAHI Alioth	Cross-sectional, descriptive, and retrospective
								85 kVp	
								6 mA	
								80 × 80 mm FOV	
60	Silva et al. ^*∗*^ [85]	Brazilian population	Brazil	154	146	America	“3 separate roots”	i-CAT	Cross-sectional, descriptive, and retrospective
								120 kVp	
								7 mA	
								Voxel size 200 *μ*m	
								FOV 80 × 80 mm	
61	Torres et al. [86]	Belgium population and Chilean population	Belgium	Belgium 100. Chile 170	145 (Belgium)	Europe and America	“Number of roots”	3D Accuitomo 170®	Cross-sectional, analytical, and retrospective
			Chile					90 kVp	
					146 (Chile)			Voxel size 0.25 mm	
62	Tredoux et al. [87]	South African population	South Africa	Patient-level was not reported	369	Africa	“Three-rooted”	Planmeca ProMax 3D Max	Cross-sectional, descriptive, and retrospective
								Voxel size 100–600 *μ*m	
								54–90 kVp	
								1–14 mA	
63	Tu et al. [88]	Taiwanese population	Taiwan	123	246	East Asia	“Extra DL”	i-CAT	Cross-sectional, descriptive, and retrospective
								Voxel size 0.2–0.4 mm	
64	Wang et al. [89]	Western Chinese population	China	558	558	East Asia	“Extra distolingual root”	3D Accuitomo	Cross-sectional, descriptive, and retrospective
								Voxel size 0.125 mm	
65	Wang et al. [90]	Mongoloid population	China	502	502	East Asia	“Three-rooted”	DCTPRO	Cross-sectional, descriptive, and retrospective
								FOV 16 × 7 cm	
								0.20 voxel size	
								90 kVp	
								9 mA	
66	Wu et al. [91]	Taiwanese population	Taiwan	233	466	East Asia	“Distolingual root”	NewTom 5G	Cross-sectional, analytical, and retrospective
								110 kVp	
								11.94 mA	
								FOV 30.5 cm^2^ × 20.3 cm^2^	
67	Wu et al. (A) [16]	Taiwanese population	Taiwan	300	600	East Asia	“Distolingual root”	NewTom 5G	Cross-sectional, analytical, and retrospective
								110 kVp	
								11.94 mA	
								FOV 30.5 cm^2^ × 20.3 cm^2^	
68	Wu et al. (B) [17]	Taiwanese population	Taiwan	400	800	East Asia	“Distolingual root”	NewTom 5G	Cross-sectional, analytical, and retrospective
								110 kVp	
								11.94 mA	
								FOV 30.5 cm^2^ × 20.3 cm^2^	
69	Xu et al. [92]	Chinese population	China	334	Tooth-level data was not reported	East Asia	“Distolingual root”	NewTom VGI	Cross-sectional, descriptive, and retrospective
								110 kVp	
								2.79 mA	
								FOV 8 cm × 8 cm	
								Voxel size 0.125 mm	
70	Zhang et al. [93]	Chinese population	China	211	232	East Asia	“Distolingual root”	3D Accuitomo	Cross-sectional, descriptive, and retrospective
								Voxel size 0.125 mm	
								5 mA	
								80 kVp	
								FOV 40 × 40 mm	
								60 × 60 mm	
71	Zhang et al. [8]	Chinese population	China	455	910	East Asia	“DL”	Galileos	Cross-sectional, descriptive, and retrospective
								85 kVp	
								35 mA	
								0.125 voxel size	
72	Zhang et al. [13]	Chinese population	China	83	83	East Asia	“Separate DL”	Galileos	Cross-sectional, descriptive, and retrospective
								85 kVp	
								28–42 mA	
								Voxel size 0.25 mm	

^*∗*^Zero prevalence of three-rooted PMFM, (*τ*): foreign language translated to English, and ($): Spiral Computed Tomography (SCT).

## Data Availability

The data that support the findings of the meta-analysis are available from the corresponding author, Nyan Min Aung, upon reasonable request.

## References

[1] Collins W. (2018). *“Collins English Dictionary”*.

[2] Shemesh A., Levin A., Katzenell V. (2015). Prevalence of 3- and 4-rooted first and second mandibular molars in the Israeli population. *Journal of Endodontics*.

[3] Kantilieraki E., Delantoni A., Angelopoulos C. (2019). Evaluation of root and root canal morphology in mandibular first and second molars in a Greek population- a CBCT study. *European Endodontic Journal*.

[4] Souza-Flamini L. E., Leoni G. B., Chaves J. F. M. (2014). The radix entomolaris and paramolaris: a micro-computed tomographic study of 3-rooted mandibular first molars. *Journal of Endodontics*.

[5] Doyle L. H., Goodell S. L., Krell G. G. (2019). Glossary of endodontic terms. *American Association of Endodontists (AAE), United State: Berman*.

[6] Duman S. B., Duman S., Bayrakdar I. S., Yasa Y., Gumussoy I. (2020). Evaluation of radix entomolaris in mandibular first and second molars using cone-beam computed tomography and review of the literature. *Oral Radiology*.

[7] Kim K. R., Song J. S., Kim S.-O., Kim S. H., Park W., Son H.-K. (2013). Morphological changes in the crown of mandibular molars with an additional distolingual root. *Archives of Oral Biology*.

[8] Zhang X., Xiong S., Ma Y. (2015). A cone-beam computed tomographic study on mandibular first molars in a Chinese subpopulation. *PLoS One*.

[9] Zanza A., Seracchiani M., Reda R. (2021). Role of the crystallographic phase of NiTi rotary instruments in determining their torsional resistance during different bending conditions. *Materials (Basel)*.

[10] Bhandi S., Mashyakhy M., Abumelha A. S. (2021). Complete obturation-cold lateral condensation vs. Thermoplastic techniques: a systematic review of micro-CT studies. *Materials*.

[11] Wang Q., Yu G., Zhou X.-d., Peters O. A., Zheng Q.-h., Huang D.-m. (2011). Evaluation of x-ray projection angulation for successful radix entomolaris diagnosis in mandibular first molars in vitro. *Journal of Endodontics*.

[12] Kim Y., Roh B.-D., Shin Y., Kim B. S., Choi Y.-l., Ha A. (2018). Morphological characteristics and classification of mandibular first molars having 2 distal roots or canals: 3-dimensional biometric analysis using cone-beam computed tomography in a Korean population. *Journal of Endodontics*.

[13] Zhang X., Xu N., Wang H., Yu Q. (2017). A cone-beam computed tomographic study of apical surgery-related morphological characteristics of the distolingual root in 3-rooted mandibular first molars in a Chinese population. *Journal of Endodontics*.

[14] Gu Y., Zhou P., Ding Y., Wang P., Ni L. (2011). Root canal morphology of permanent three-rooted mandibular first molars: part III-an odontometric analysis. *Journal of Endodontics*.

[15] Huang R.-Y., Lin C.-D., Lee M.-S. (2007). Mandibular disto-lingual root: a consideration in periodontal therapy. *Journal of Periodontology*.

[16] Wu Y.-C., Cheng W.-C., Chung M.-P. (2018). Complicated root canal morphology of mandibular lateral incisors is associated with the presence of distolingual root in mandibular first molars: a cone-beam computed tomographic study in a Taiwanese population. *Journal of Endodontics*.

[17] Wu Y.-C., Cathy Tsai Y.-W., Cheng W.-C. (2018). Relationship of the incidence of C-shaped root canal configurations of mandibular first premolars with distolingual roots in mandibular first molars in a Taiwanese population: a cone-beam computed tomographic study. *Journal of Endodontics*.

[18] Curzon M. E. J. (1973). Three-rooted mandibular permanent molars in English Caucasians. *Journal of Dental Research*.

[19] Chandra S. S., Chandra S., Shankar P., Indira R. (2011). Prevalence of radix entomolaris in mandibular permanent first molars: a study in a South Indian population. *Oral Surgery, Oral Medicine, Oral Pathology, Oral Radiology & Endodontics*.

[20] Magnucki G., Mietling S. V. K. (2021). Four-rooted maxillary first molars: a systematic review and meta-analysis. *International Journal of Dentistry*.

[21] Wolf T. G., Anderegg A. L., Wierichs R. J., Campus G. (2021). Root canal morphology of the mandibular second premolar: a systematic review and meta-analysis. *BMC Oral Health*.

[22] Wolf T. G., Anderegg A. L., Yilmaz B., Campus G. (2021). Root canal morphology and configuration of the mandibular canine: a systematic review. *International Journal of Environmental Research and Public Health*.

[23] Kottoor J., Albuquerque D., Velmurugan N., Kuruvilla J. (2013). Root anatomy and root canal configuration of human permanent mandibular premolars: a systematic review. *Anatomy Research International*.

[24] de Pablo Ó. V., Estevez R., Péix Sánchez M., Heilborn C., Cohenca N. (2010). Root anatomy and canal configuration of the permanent mandibular first molar: a systematic review. *Journal of Endodontics*.

[25] Leal Silva E. J. N., Pradob M. C., Hungaro Duartec M. A. (2021). Prevalence of root canal system configurations in the Brazilian population analyzed by cone-beam computed tomography – a systematic review. *Revista Portuguesa de Estomatologia, Medicina Dentária e Cirurgia Maxilofacial*.

[26] Khurayzi T. A., Beleges E. M., Dallak S. A. (2021). The prevalence of radix entomolaris (RE) in the mandibular permanent first molars among the Saudi arabian population–a systematic review. *Saudi Journal of Oral Dental Research*.

[27] Tomaszewska I. M., Skinningsrud B., Jarzębska A., Pękala J. R., Tarasiuk J., Iwanaga J. (2018). Internal and external morphology of mandibular molars: an original micro-CT study and meta-analysis with review of implications for endodontic therapy. *Clinical Anatomy*.

[28] Saletta J. M., Garcia J. J., Caramês J. M. M., Schliephake H., da Silva Marques D. N. (2019). Quality assessment of systematic reviews on vertical bone regeneration. *International Journal of Oral and Maxillofacial Surgery*.

[29] Furuya-Kanamori L., Barendregt J. J., Doi S. A. R. (2018). A new improved graphical and quantitative method for detecting bias in meta-analysis. *International Journal of Evidence-Based Healthcare*.

[30] Abarca J., Duran M., Parra D., Steinfort K., Zaror C., Monardes H. (2020). Root morphology of mandibular molars: a cone-beam computed tomography study. *Folia Morphologica*.

[31] AL-Alawi H., Al-Nazhan S., Al-Maflehi N. (2019). The prevalence of radix molaris in the mandibular first molars of a Saudi subpopulation based on cone-beam computed tomography. *Restorative Dentistry and Endodontics*.

[32] Albarzanji H., Muhamed Jamil A. (2020). The variation in the number of roots and canals morphology of permanent mandibular first molar teeth by using cone beam computed tomography imaging in a sample of Erbil city. *Erbil Dental Journal*.

[33] Alenezi K., Alharbi B., Kolarkodi S. (2020). Prevalence of three rooted mandibular permanent first molars in Qassim population in Saudi Arabia. *International Journal of Medicine in Developing Countries*.

[34] Atsushi O., Shotaro S. (2017). Evaluation of root anatomy and root canal configurations of mandibular first molars using dental cone-beam computed tomography. *Nippon Endodontic Magazine*.

[35] Bagherpour A., Jafarzadeh H., Raeeis-Sattari F. (2020). Morphologic evaluation of the prevalence of radix root and mid-mesial canal in the mandibular first molars using CBCT during 2016-2018 in patients referred to mashhad dental school. *Journal of Mashhad Dental School*.

[36] Caputo B. V., Filho G. A. N., Salgado D. M. R. de A. (2016). Evaluation of the root canal morphology of molars by using cone-beam computed tomography in a Brazilian population: Part I. *Journal of Endodontics*.

[37] Celikten B., Tufenkci P., Aksoy U. (2016). Cone beam CT evaluation of mandibular molar root canal morphology in a Turkish Cypriot population. *Clinical Oral Investigations*.

[38] Chakradhar A., Nepal M., Pradhan S. (2021). Occurrence of extra roots in permanent mandibular molars: a cone beam computed tomography study. *Journal of Nepalese Society of Periodontology and Oral Implantology*.

[39] Choi M.-R., Moon Y.-M., Seo M.-S. (2015). Prevalence and features of distolingual roots in mandibular molars analyzed by cone-beam computed tomography. *Imaging Science in Dentistry*.

[40] Dastgerdi A. C., Navabi M., Hafezi L. (2018). Anatomy of permanent mandibular first molars in a selected Iranian population using cone-beam computed tomography. *Iranian Endodontic Journal*.

[41] Demirbuga S., Sekerci A., Dincer A., Cayabatmaz M., Zorba Y. (2013). Use of cone-beam computed tomography to evaluate root and canal morphology of mandibular first and second molars in Turkish individuals. *Medicina Oral, Patología Oral y Cirugía Bucal*.

[42] Deng P. U., Halim M. S., Masudi S. a. M., Al-Shehadat S., Ahmad B. (2018). Cone-beam computed tomography analysis on root and canal morphology of mandibular first permanent molar among multiracial population in East Coast Malaysian population. *European Journal of Dermatology*.

[43] Estrela C., Bueno M. R., Couto G. S. (2015). Study of root canal anatomy in human permanent teeth in A subpopulation of Brazil’s center region using cone-beam computed tomography - Part 1. *Brazilian Dental Journal*.

[44] Felsypremila G., Vinothkumar T. S., Kandaswamy D. (2015). Anatomic symmetry of root and root canal morphology of posterior teeth in Indian subpopulation using cone beam computed tomography: a retrospective study. *European Journal of Dermatology*.

[45] Kumar Garg A., Tewari R. K., Agrawal N. (2013). Prevalence of three-rooted mandibular first molars among Indians using SCT. *International Journal of Dentistry*.

[46] Guo J., Vahidnia A., Sedghizadeh P. (2015). Root and canal morphology of mandibular permanent first molars in a US population – a multi-ethnicity evaluation by CBCT. *ENDO*.

[47] Hai-feng M., Hai-xia M., Jun-rong Q. (2017). Variations in the root and root canal of permanent mandibular first molars in the Han Population of southwest Shandong Province: a three-dimensional reconstruction based on cone-beam CT data using Planmeca Romexis software. *Chinese Journal of Tissue Engineering Research*.

[48] Hiran-us S., Benjavongkulchai S., Antanit C. (2021). Prevalence of C-shaped canals and three-rooted mandibular molars using CBCT in a selected Thai population. *Iranian Endodontic Journal*.

[49] Hosseini S., Soleymani A., Moudi E. (2020). Frequency of middle mesial canal and radix entomolaris in mandibular first molars by cone beam computed tomography in a selected Iranian population. *Caspian Journal of Dental Research*.

[50] Huang C.-C., Chang Y.-C., Chuang M.-C. (2010). Evaluation of root and canal systems of mandibular first molars in Taiwanese individuals using cone-beam computed tomography. *Journal of the Formosan Medical Association*.

[51] Jamshidi D., Tofangchiha M., Roohi N. (2021). Evaluation of the association between the number and configuration of root canals of mandibular molars in an Iranian subpopulation: a cone-beam computed tomography study. *Saudi Endodontic Journal*.

[52] Jang J.-K., Peters O. A., Lee W., Son S.-A., Park J.-K., Kim H.-C. (2013). Incidence of three roots and/or four root canals in the permanent mandibular first molars in a Korean sub-population. *Clinical Oral Investigations*.

[53] Jang Y. E., Kim Y., Kim B., Kim S. Y, Kim H. J (2019). Frequency of non-single canals in mandibular premolars and correlations with other anatomical variants: an in vivo cone beam computed tomography study. *BMC Oral Health*.

[54] Jayasinghe R. D., Li T. K. L. (2007). Three-rooted first permanent mandibular molars in a Hong Kong Chinese population: a computed tomographic study. *Hong Kong Dental Journal*.

[55] Kaya Buyukbayran Ö. Ü. I., Çolakoglu Ö. Ü. G., Elcin D. M. A. (2021). Evaluation of Radix Entomolaris, Radix Paramolaris and C-shaped canals in mandibular molars using cone-beam computed tomography. *The Journal of the Dental Faculty of Ankara University*.

[56] Kim S.-Y., Yang S.-E. (2012). Cone-beam computed tomography study of incidence of distolingual root and distance from distolingual canal to buccal cortical bone of mandibular first molars in a Korean population. *Journal of Endodontics*.

[57] Kim S., Choi M.-R., Yoo J.-J. (2013). Concurrent relationship between additional canals of mandibular first molars and maxillary first molars using cone-beam computed tomography. *Oral Radiology*.

[58] Kim S.-Y., Kim B. S., Woo J., Kim Y. (2013). Morphology of mandibular first molars analyzed by cone-beam computed tomography in a Korean population: variations in the number of roots and canals. *Journal of Endodontics*.

[59] Kim H. H., Jo H. H., Min J. B., Hwang H. K (2018). CBCT study of mandibular first molars with a distolingual root in Koreans. *Restorative dentistry & endodontics*.

[60] Martins J. N. R., Marques D., Mata A., Caramês J. (2017). Root and root canal morphology of the permanent dentition in a Caucasian population: a cone-beam computed tomography study. *International Endodontic Journal*.

[61] Martins J. N. R., Gu Y., Marques D. (2018). Differences on the root and root canal morphologies between asian and white ethnic groups analyzed by cone-beam computed tomography. *Journal of Endodontics*.

[62] Mashyakhy M., Gambarini G. (2019). Root and root canal morphology differences between genders: a comprehensive in-vivo CBCT study in a Saudi population. *Acta Stomatologica Croatica*.

[63] Alamir A., Mashyakhy M., Renugalakshmi A. (2021). Ethnical anatomical differences in mandibular first permanent molars between Indian and Saudi arabian subpopulations: a retrospective cross-sectional study. *The Journal of Contemporary Dental Practice*.

[64] Miloglu O., Arslan H., Barutcigil C., Cantekin K. (2013). Evaluating root and canal configuration of mandibular first molars with cone beam computed tomography in a Turkish population. *Journal of Dental Science*.

[65] Monsarrat P., Arcaute B., Peters O. A. (2016). Interrelationships in the variability of root canal anatomy among the permanent teeth: a full-mouth approach by cone-beam CT. *PLoS One*.

[66] Nazeer M. R., Khan F. R. (2019). Evaluation of the root and canal morphology of mandibular first permanent molars in a sample of Pakistani population by cone-beam computed tomography. *JPMA. The Journal of the Pakistan Medical Association*.

[67] Ngeow W. C., Redzuan N. R., Mat Nawawi N. N. A. (2020). A cone-beam computed tomography study on the morphometry of the mandibular molars and their relative root lengths to the mandibular height. *Archives of Orofacial Sciences*.

[68] Ni N., Cao S., Han L., Zhang L., Ye J., Zhang C. (2018). Cone-beam computed tomography analysis of root canal morphology in mandibular first molars in a Chinese population: a clinical study. *Evidence-Based Endodontics*.

[69] Nur B. G., Ok E., Altunsoy M., Aglarci O. S., Colak M., Gungor E. (2014). Evaluation of the root and canal morphology of mandibular permanent molars in a south-eastern Turkish population using cone-beam computed tomography. *European Journal of Dermatology*.

[70] Pan J. Y. Y., Parolia A., Chuah S. R., Bhatia S., Mutalik S., Pau A. (2019). Root canal morphology of permanent teeth in a Malaysian subpopulation using cone-beam computed tomography. *BMC Oral Health*.

[71] Park J. B., Kim N., Park S., Kim Y., Ko Y. (2013). Evaluation of root anatomy of permanent mandibular premolars and molars in a Korean population with cone-beam computed tomography. *European Journal of Dermatology*.

[72] Patil S., Yadav N., Al-Zoubi I. (2018). Three-rooted mandibular first molars in a Saudi arabian population: a CBCT study. *Pesquisa Brasileira em Odontopediatria e Clínica Integrada*.

[73] Perez-Heredia M., Ferrer-Luque C. M., Bravo M. (2017). Cone-beam computed tomographic study of root anatomy and canal configuration of molars in a Spanish population. *Journal of Endodontics*.

[74] Pham K., Le A. L. (2019). Evaluation of roots and canal systems of mandibular first molars in a Vietnamese subpopulation using cone-beam computed tomography. *Journal of International Society of Preventive and Community Dentistry*.

[75] Plotino G., Tocci L., Grande N. M. (2013). Symmetry of root and root canal morphology of maxillary and mandibular molars in a white population: a cone-beam computed tomography study in vivo. *Journal of Endodontics*.

[76] Popovic M., Zivanovic S., Vucicevic T., Grujovic M., Papic M. (2020). Cone-beam computed tomography study of tooth root and canal morphology of permanent molars in a Serbian population. *Vojnosanitetski Pregled*.

[77] Qiao X., Zhu H., Yan Y. (2020). Prevalence of middle mesial canal and radix entomolaris of mandibular first permanent molars in a western Chinese population: an in vivo cone-beam computed tomographic study. *BMC Oral Health*.

[78] Razumova S., Brago A., Khaskhanova L. (2018). A cone-beam computed tomography scanning of the root canal system of permanent teeth among the moscow population. *International Journal of Dentistry*.

[79] Riyahi A. M., Alssum K., Hadadi H., Alsayyari A., Alebrah T., Aljarbou F. (2019). Prevalence of three-rooted mandibular permanent first and second molars in the Saudi population. *The Saudi Dental Journal*.

[80] Rahimi S., Mokhtari H., Ranjkesh B. (2017). Prevalence of extra roots in permanent mandibular first molars in Iranian population: a CBCT analysis. *Iranian Endodontic Journal*.

[81] Rodrigues C. T., Oliveira-Santos C. d., Bernardineli N. (2016). Prevalence and morphometric analysis of three-rooted mandibular first molars in a Brazilian subpopulation. *Journal of Applied Oral Science*.

[82] Senan E., Madfa A. A., Alhadainy H. A. (2020). Root and canal configuration of mandibular first molars in a Yemeni population: a cone-beam computed tomography. *European Endodontic Journal*.

[83] Sharaan M., Elrawdy A. (2017). An evaluation of mandibular molars root canal morphology using cone-beam computed tomography in an Egyptian subpopulation. *Tanta Dental Journal*.

[84] Shehadat S. A., Waheb S., Bayatti S. W. A. (2019). Cone beam computed tomography analysis of root and root canal morphology of first permanent lower molars in a Middle East subpopulation. *Journal of International Society of Preventive and Community Dentistry*.

[85] Silva E. J. N. L., Nejaim Y., Silva A. V., Haiter-Neto F., Cohenca N. (2013). Evaluation of root canal configuration of mandibular molars in a Brazilian population by using cone-beam computed tomography: an in vivo study. *Journal of Endodontics*.

[86] Torres A., Jacobs R., Lambrechts P. (2015). Characterization of mandibular molar root and canal morphology using cone beam computed tomography and its variability in Belgian and Chilean population samples. *Imaging Science in Dentistry*.

[87] Tredoux S., Warren N., Buchanan G. D. (2021). Root and canal configurations of mandibular first molars in a South African subpopulation. *Journal of Oral Science*.

[88] Tu M.-G., Huang H.-L., Hsue S.-S. (2009). Detection of permanent three-rooted mandibular first molars by cone-beam computed tomography imaging in Taiwanese individuals. *Journal of Endodontics*.

[89] Wang Y., Zheng Q.-h., Zhou X.-d. (2010). Evaluation of the root and canal morphology of mandibular first permanent molars in a western Chinese population by cone-beam computed tomography. *Journal of Endodontics*.

[90] Wang X., Zhang Y., Li X. (2019). Biometric analysis of apical surgery-related anatomy of mandibular first molars: a cone-beam computed tomography study in a Mongoloid population. *Journal of International Medical Research*.

[91] Wu Y. C., Su C. C., Tsai Y. W. C. (2017). Complicated root canal configuration of mandibular first premolars is correlated with the presence of the distolingual root in mandibular first molars: a cone-beam computed tomographic study in Taiwanese individuals. *Journal of Endodontics*.

[92] Xu S., Dao J., Liu Z., Zhang Z., Lu Y., Zeng X. (2020). Cone-beam computed tomography investigation of middle mesial canals and isthmuses in mandibular first molars in a Chinese population. *BMC Oral Health*.

[93] Zhang R., Wang H., Tian Y.-Y., Yu X., Hu T., Dummer P. M. H. (2011). Use of cone-beam computed tomography to evaluate root and canal morphology of mandibular molars in Chinese individuals. *International Endodontic Journal*.

[94] Schäfer E., Breuer D., Janzen S. (2009). The prevalence of three-rooted mandibular permanent first molars in a German population. *Journal of Endodontics*.

[95] Curzon M. E. (1974). Miscegenation and the prevalence of three-rooted mandibular first molars in the Baffin Eskimo. *Community Dentistry and Oral Epidemiology*.

[96] Gulabivala K., Aung T. H., Alavi A., Ng Y.-L. (2001). Root and canal morphology of Burmese mandibular molars. *International Endodontic Journal*.

[97] Reichart P. A., Metah D. (1981). Three-rooted permanent mandibular first molars in the Thai. *Oral Epidemiology*.

[98] Sperber G. H., Moreau J. L. (1998). Study of the number of roots and canals in Senegalese first permanent mandibular molars. *International Endodontic Journal*.

[99] Younes S. A., Al-Shammery A. R., El-Angbawi M. F. (1990). Three-rooted permanent mandibular first molars of Asian and black groups in the Middle East. *Oral Surgery, Oral Medicine, Oral Pathology*.

[100] Australian Human Rights Commission (2018). Leading for Change, A blueprint for cultural diversity and inclusive leadership revisited.

[101] Martins J. N. R., Nole C., Ounsi H. F. (2021). Worldwide assessment of the mandibular first molar second distal root and root canal: a cross-sectional study with meta-analysis. *Journal of Endodontics*.

[102] Munn Z., Moola S., Lisy K., Riitano D., Tufanaru C. (2015). Methodological guidance for systematic reviews of observational epidemiological studies reporting prevalence and cumulative incidence data. *International Journal of Evidence-Based Healthcare*.

